# A Contemporary Review of Blood Transfusion in Critically Ill Patients

**DOI:** 10.3390/medicina60081247

**Published:** 2024-07-31

**Authors:** Sumeet K. Yadav, Guleid Hussein, Bolun Liu, Nikhil Vojjala, Mohamed Warsame, Mohamad El Labban, Ibtisam Rauf, Mohamed Hassan, Tashfia Zareen, Syed Muhammad Usama, Yaqi Zhang, Shika M. Jain, Salim R. Surani, Pavan Devulapally, Brian Bartlett, Syed Anjum Khan, Nitesh Kumar Jain

**Affiliations:** 1 Department of Hospital Internal Medicine, Mayo Clinic Health System, 1025 Marsh Street, Mankato, MN 56001, USA; yadav.sumeet@mayo.edu (S.K.Y.); hussein.guleid@mayo.edu (G.H.); liu.bolun@mayo.edu (B.L.); warsame.mohamed@mayo.edu (M.W.); hassan.mohamed2@mayo.edu (M.H.); 2 Department of Internal Medicine, Trinity Health Oakland/Wayne State University, Pontiac, MI 48341, USA; vojjalanikhil@gmail.com; 3 Department of Internal Medicine, Mayo Clinic Health System, 1025 Marsh Street, Mankato, MN 56001, USA; ellabban.mohamad@mayo.edu; 4 St. George’s University School of Medicine, St. George SW17 0RE, Grenada; ibi.rauf@gmail.com (I.R.); tashfiaz@gmail.com (T.Z.); 5 Department of Internal Medicine, Nazareth Hospital, Philadelphia, PA 19152, USA; syedmuhammad.usama@mercyhealth.org; 6 Department of Internal Medicine, University of Iowa Hospitals and Clinics, Iowa City, IA 52242, USA; yaqi920928@gmail.com; 7 Department of Internal Medicine, MVJ Medical College and Research Hospital, Bengaluru 562 114, India; shikha.jain1885@gmail.com; 8 Department of Medicine and Pharmacology, Texas A&M University, College Station, TX 79016, USA; 9 South Texas Renal Care Group, Department of Nephrology, Christus Santa Rosa, Methodist Hospital, San Antonio, TX 78229, USA; dpavan2000@yahoo.com; 10 Department of Emergency Medicine, Mayo Clinic health System, 1025 Marsh Street, MN 56001, USA; bartlett.brian@mayo.edu; 11 Department of Critical Care Medicine, Mayo Clinic Health System, 1025 Marsh Street, Mankato, MN 56001, USA; khan.syed@mayo.edu

**Keywords:** transfusion, coagulopathy, critically ill, guideline, intensive care, plasma, platelets, red blood cells, point of care, tranexamic acid, bleeding, coagulation, massive transfusion, anemia, platelet transfusion, plasma, hemostasis, adult, thrombocytopenia, coagulopathy, prophylactic transfusion, blood, cardiac surgery, acute coronary syndrome, erythrocyte, hemoglobin, practice, risk factors, surgery, transfusion

## Abstract

Blood transfusion is a common therapeutic intervention in hospitalized patients. There are numerous indications for transfusion, including anemia and coagulopathy with deficiency of single or multiple coagulation components such as platelets or coagulation factors. Nevertheless, the practice of transfusion in critically ill patients has been controversial mainly due to a lack of evidence and the need to consider the appropriate clinical context for transfusion. Further, transfusion carries many risk factors that must be balanced with benefits. Therefore, transfusion practice in ICU patients has constantly evolved, and we endeavor to present a contemporary review of transfusion practices in this population guided by clinical trials and expert guidelines.

## 1. Introduction

The history of blood transfusion dates back to 43 BC in the seventh book of “Metamorphoses”, and the first transfusion in humans was documented in the 1800s by a British surgeon [1]. Since then, blood transfusion has been used as a life-saving measure, especially in those with blood loss [2]. With the advancement in medicine, there has been an exponential rise in blood and blood product transfusion for several medical conditions. Anemia, thrombocytopenia, and coagulation disorders are some of the common reasons for blood transfusion [3]. While transfusion has benefits, it does come with well-known risks and complications. Transfusion-related reactions, acute lung injury, iron overload, and alloimmunization are not unfamiliar among physicians (Table 1) [3,4]. Hence, physicians need to familiarize themselves with thresholds to keep transfusion balanced with benefits. The need to transfuse is often a matter of judgment in the right clinical context.

Patients admitted to the intensive care unit (ICU) are critically ill compared to those admitted to hospital floors. It is estimated that about 95% of the patients will be anemic by Day 3 of their ICU stay, and this anemia will persist throughout their stay with or without transfusion [5,6,7]. About forty to fifty percent of critically ill patients require at least one unit of red blood cell (RBC) transfusion during their stay, and it increases significantly with prolonged admission [8]. The practice of transfusion in critically ill patients has been controversial mainly due to a lack of evidence and the need to consider the appropriate clinical context for transfusion. Preventive strategies such as the use of small-volume phlebotomy tubes for diagnostic testing and the adoption of closed-loop systems have been shown to reduce transfusion requirements [5].

Guidelines for transfusion that may have been developed for non-critically ill patients may not be enough to justify transfusion recommendations in critically ill patients with hemodynamic instability. Several trials have been published for transfusion in critically ill patients but with inconsistent recommendations on whether to utilize restrictive transfusion, i.e., hemoglobin (Hb) level of 7 to 8 g/dL, or to liberalize transfusion beyond these conservative levels [6,7,8,9]. This study aims to provide a contemporary review of current evidence for the transfusion of blood and blood products with their indication and transfusion threshold in critically ill patients, summarizing both clinical trials and expert guidelines. We endeavor to provide a transfusion-related working guide for clinicians working with critically ill patients in the ICU.

## 2. Red Blood Cell Transfusion

Red blood cell transfusion can be a lifesaving intervention for patients with severe anemia and hemorrhagic shock [2]. However, the threshold of RBC transfusion in critically ill patients is unclear in patients with moderate anemia (Hb 7 to 10 gm/dL).

Physiologically, hemoglobin is needed to deliver oxygen to the tissue and prevent hypoxia. Hypoxia is known to induce vasodilation due to nitric oxide release, which reduces vascular resistance and afterload [10]. A normal cardiovascular compensation would be increased preload and cardiac output by increasing the stroke volume and heart rate [11,12]. However, critically ill patients have limitations of compensatory mechanisms with impaired cardiac output response to anemia, leading to heart failure and increased cardiac morbidity and mortality [13]. Several critically ill patients also have respiratory failure with increased work of breathing, which can decrease the oxygen content of the blood [14]. Sepsis can cause microcirculatory dysfunction, impairing oxygen availability at the cellular level by decreasing oxygen extraction [15,16]. Using vasoactive agents may interfere with the physiological adjustments in regional blood flow. Sedation can impair physiologic cardiovascular adjustments to anemia by depressing cardiac function, altering vascular tone, and blunting the autonomic responses [17]. Blood transfusion improves hemodynamics in critically ill patients by optimizing pre-load, increasing circulatory volume and hemoglobin, increasing the oxygen-carrying capacity of the blood, mitigating vasopressors’ needs, and improving blood flow autoregulation to the tissues [18,19].

RBC transfusion in critically ill patients should be a decision based on the patient’s comprehensive clinical picture rather than an arbitrary lab value. With more emerging data from randomized trials, current practice tends to favor more restrictive strategies rather than targeting hemoglobin from a physiological standpoint alone, indicating that many other factors play a crucial role in deciding overall Benefit vs. morbidity/mortality risk in these critically ill patients [20]. The landmark “Transfusion Requirements in Critical Care (TRICC)” trial, published in 1999, remains the largest randomized controlled clinical trial investigating the impact of transfusion in non-bleeding patients on outcomes in adult critically ill patients and allowing us to analyze the direct effect of transfusion and the consequences of other confounders associated with transfusion requirement [8]. As per this landmark study, RBC transfusion did not offer any survival advantage in euvolemic patients without active bleeding when hemoglobin exceeded seven g/dL and oxygen content was increased by 20% following transfusion. Since then, many trials have compared restrictive vs. liberal transfusion strategies, thus guiding societal recommendations of clinical practice guidelines among these patients. The three critical trials on transfusion are the “TRICC” trial, the “TRISS” trial performed in patients with septic shock, and the “RELIEVE” trial performed in patients with mechanical ventilation [7,8,9]. All three trials found no difference in the overall mortality favoring restrictive transfusion strategies. Additional pertinent details of these three randomized clinical trials are available in Table 2.

Based on the current evidence shown in Table 2, there is no significant difference in survival outcome between the restrictive and liberal strategies for critically ill patients with septic shock and those mechanically ventilated. Several societies have their own guidelines for transfusion based on their clinical conditions, as shown in Table 3. Physicians could utilize hemodynamic factors to determine whether restrictive or liberal strategies should be considered.

### 2.1. Evidence of Transfusion in Septic Shock

Sepsis-induced shock is characterized by a dysregulated immune response to infection, leading to organ dysfunction and circulatory collapse. In septic shock, microcirculatory dysfunction and impaired oxygen utilization often necessitate the consideration of blood transfusion [15]. Current guidelines, such as those from the Surviving Sepsis Campaign, recommend a restrictive approach (Hb less than 7 g/dL) to transfusion in septic shock, which is based on the Transfusion Requirements in Septic Shock (TRISS) and Transfusion Requirements in Critical Care (TRICC) trial [7,26]. The TRISS trial randomly assigned patients in the ICU who had septic shock and compared RBC transfusion between Hb < 7 g/dL and Hb < 9 g/dL, and there was no significant difference in survival, use of organ support, and ischemia at three months [7]. Similarly, in a meta-analysis evaluating transfusion in septic shock patients in critical care settings, restrictive vs. liberal transfusion did not differ in terms of mortality, but increased transfusion was associated with increased nosocomial infection, acute lung, and acute kidney injury [27]. In another study with patients who already had acute kidney injury due to sepsis, restrictive transfusion did not affect survival but created a prolonged length of hospital stay [28]. In the elderly population (>65 years) and sepsis, transfusion when Hb < 10 g/dL was associated with decreased mortality [29].

### 2.2. Evidence of RBC-Transfusion Strategies in Patients with Acute Myocardial Infarction

Patients with coronary artery disease (CAD) frequently require intensive care unit (ICU) stays, with acute coronary syndrome (ACS) being one of the most common critical conditions encountered. ACS is precipitated by acute thrombosis and/or critical stenosis of the coronary arteries, leading to diminished myocardial blood flow [24]. It is not exceedingly rare to see patients with ACS having concomitant anemia. The hypothesis of improved oxygenation due to RBC transfusion in these patients, thereby improving outcomes, is counteracted by mechanisms such as RBC-induced platelet activation and vasoconstriction caused by decreased nitric oxide levels [30]. Several RCTs investigated the RBC-transfusion threshold in ACS patients. The CRIT pilot study included 45 patients with acute myocardial infarction (AMI) and hematocrit level ≤ 30%, with randomization to a liberal group (transfuse if hematocrit < 30% to the goal of 30–33%) or a conservative group (transfuse if hematocrit < 24% to the goal of 24–27%). The primary clinical safety measurement of in-hospital death, recurrent MI, or new or worsening congestive heart failure occurred more in the liberal transfusion group, suggesting that more transfusion may be associated with poorer clinical outcomes [31]. The REALITY trial investigated one-year major cardiovascular events after restrictive (transfusion triggered by hemoglobin ≤ 8 g/dL, target between 8 and 10 g/dL) versus liberal (transfusion triggered by hemoglobin ≤ 10 g/dL, target > 11 g/dL) blood-transfusion strategies in patients with AMI. The results suggested that the restrictive arm achieved noninferiority at 30 days but not at the 1-year analysis [32]. A systematic review and meta-analysis combined the subset analysis of AMI patients in the TRICS-III trial and concluded that liberal strategies may improve patient outcomes [33,34]. Hence, a schematically designed clinical trial is of paramount importance and was an unmet need to address this research question. The Myocardial Ischemia and Transfusion (MINT) trial and MINT investigators attempted to answer this unsolved enigma without bias. The MINT trial included 3504 patients with STEMI or NSTEMI with concomitant anemia and compared the effect of a liberal (<10 g/dL) vs. restrictive blood (7 or 8 g/dL) transfusion strategy. The trial did not find a liberal transfusion strategy targeting Hgb concentration ≥ 10 g/dL to be superior to a restrictive target of 7–8 g/dL regarding all-cause death or nonfatal MI at 30 days [35].

For stable coronary artery disease patients who require an ICU stay, limited data recommended a higher transfusion threshold (less than 8 g/dL) [36], and current guidelines maintain higher transfusion thresholds [23,35,37]. It is well known that anemia occurs commonly in chronic heart failure patients, but the clinical utility of blood transfusion is controversial. Thus, transfusion may be considered only in acute settings for severe anemia in heart failure patients [38]. There are scarce specific data about platelet or plasma transfusion in CAD or HF patients. A study investigated the impact of thrombocytopenia (platelet count < 100 × 10^9^/L) on patients undergoing percutaneous coronary intervention and found no significant difference in the composite primary outcome, including in-hospital death, bleeding events, and post-PCI transfusion [39]. Therefore, general transfusion thresholds of platelet or plasma are recommended in non-bleeding, critically ill patients with CAD or HF [24].

### 2.3. Evidence of Transfusion in Cardiac Surgery Patients

It has been reported that more than 50% of patients undergoing cardiac surgery received at least one RBC transfusion [40]. Multiple RCTs have been performed but with conflicting outcomes. The TRACS study enrolled 503 cardiac surgery patients with cardiopulmonary bypass, and there were no significant differences in 30-day mortality or severe mortality between restrictive (hematocrit 24%) and liberal (hematocrit 30%) groups [41]. The TITRe2 trial enrolled 2003 nonemergent cardiac surgery patients and found higher mortality in the restrictive group (hemoglobin < 7.5 g/dL) than in the liberal group (hemoglobin < 9 g/dL) [42]. The most recent trial, TRICS-III, was the largest RCT, involving 5243 cardiac surgery patients, and the results showed that the restrictive strategy (hemoglobin < 7.5 g/dL) was non-inferior to a liberal strategy (hemoglobin < 9.5 g/dL) [15], which was reflected in the most updated guidelines, where the restrictive transfusion strategy (with an RBC transfusion threshold of 7.5 g/dL) is recommended in non-bleeding anemic, critically ill post-cardiac surgery patients [24,37]. Observational studies on platelet transfusion following cardiac surgery have been numerous, and a recent meta-analysis indicated that platelet transfusion was not associated with perioperative complications [43]. For plasma transfusion in cardiac surgery patients, a Cochrane meta-analysis included 15 trials to investigate the efficacy of the prophylactic use of fresh frozen plasma (FFP) to prevent bleeding in cardiac surgery, the result of which revealed insufficient evidence of safety or efficacy [44]. Overall, general platelet and plasma transfusion recommendations are currently applied to critically ill cardiac surgery patients [24].

## 3. Fresh Frozen Plasma and Cryoprecipitate Transfusion

### 3.1. Fresh Frozen Plasma 

Fresh frozen plasma (FFP) is the liquid part of whole blood, frozen within 8 h of collection to less than −30° Centigrade [45]. FFP is devoid of platelets but contains all coagulation factors and other proteins present in the blood. Erythrocytes and leukocytes are also notably absent. If the plasma is frozen within 24 h but beyond 8 h, it is called frozen plasma, also called “F24” in the United States. The levels of labile factors like Factor 5 and Factor 8 may be depleted if the time to freeze plasma is prolonged [46]. A unit of FFP is 200–300 mL in volume, and 10–20 mL/kg, or approximately 4 to 6 units in adults, can increase factor levels up to 20% [47]. The goal is to achieve hemostasis with coagulation factors between 25 and 30% of normal and fibrinogen no less than 75 to 100 mg/dL [48].

The question of FFP transfusion comes into the picture in a critically ill patient in three situations: (1) prophylactic transfusion in a non-bleeding patient, not planned for any procedures; (2) prophylactic transfusion in a non-bleeding patient before performing procedures; (3) therapeutic transfusion in a bleeding patient. Currently, there is conflicting evidence on whether the correction of single lab parameters like PT/INR can correlate and predict the complex hemostatic mechanisms that occur in vivo [49]. A task force of thirteen international experts and three methodologists developed an evidence-based clinical practice guideline in 2020 regarding transfusion in non-bleeding critically ill patients [50]. They sourced six RCTs in post-cardiac surgery patients and an observational study in the general ICU population to guide the recommendations. They recommended against prophylactic frozen plasma (FP) transfusion in non-bleeding critically ill patients with coagulopathy. A significant quantity of FFP is transfused prophylactically to non-bleeding patients with coagulopathy [51]. Regarding prophylactic plasma transfusion before the procedure, the task force examined two randomized controlled trials. These trials showed no significant difference in major bleeding events and a short-term survival advantage. Notably, both trials were terminated prematurely secondary to slow recruitment, so the reduction in mortality was attributed to chance secondary to the small number of events appreciated in addition to the trials’ abrupt termination [50]. Multiple other RCTs have failed to show a clear benefit of FP transfusion before invasive procedures in reducing mortality and bleeding; however, in the analyzed studies, low to moderate doses of FP were used [51,52]. Nevertheless, complications secondary to procedures and critical in patients with coagulopathy are rare, suggesting that even if FP is effective, its impact is likely minimal [51]. In the largest multicenter prospective study conducted in the United Kingdom in 29 ICUs with over 1923 admissions, 12.7% received FFP over four weeks of ICU admission. They found a sizable portion, 31%, of the FFP treatments administered to patients with a normal or near normal prothrombin time, and 41% were given to patients without bleeding and slightly abnormal international normalized ratios with INRs of less than 2.5. Post-transfusion corrections remained minimal unless pre-transfusion INR was greater than 2.5. The study also revealed a wide variation in FFP use among ICU clinicians, raising concerns about the uncertain clinical benefits of many FFP transfusions in critically ill patients [53]. The use of FFP is unnecessarily high in the United States despite the presence of consensus guidelines [54]. 

According to the British Committee for Standards in Hematology, FFP can replace single coagulation factor deficiencies, primarily factor five, when no fractionated product is available. It can also be administered in cases of multiple coagulation factor deficiencies, disseminated intravascular coagulation, and severe bleeding with platelets [45,55]. In thrombotic thrombocytopenic purpura, prompt single-volume daily plasma exchange is recommended and is continued for at least 2 days post-remission. For patients with liver disease and prolonged prothrombin time, FFP infusion can be considered. FFP can also be used in cases of surgical bleeding and massive transfusion; however, it should be guided by time-need coagulation tests as well as regular patient assessments [55].

In summary, FFP transfusion is considered appropriate for patients with trauma requiring massive blood transfusion (1:1:1 ratio for RBC:Plasma:Platelet); hepatic disease with bleeding including DIC (with an emphasis on treating underlying cause); plasma exchange in patients who are at high risk of bleeding, like in those undergoing invasive procedures or immune-related indication such as in immune TTP/ADAMTS13 deficiency; anticoagulation related bleeding such as warfarin use when prothrombin complex concentrate (PCC) is unavailable; and bleeding associated with a single factor deficiency when the specific factor concentrate is not available [50,56,57,58]. Very specifically, prevalent practices of plasma transfusion abuse should be avoided, especially when there is a lack of bleeding or to correct an abnormal coagulation test [45].

Several risks are associated with FFP transfusion. Transfusion-related acute lung injury (TRALI), transfusion-associated circulatory overload (TACO), and allergic/anaphylactic reactions are significant. Other less common risks include infections, febrile nonhemolytic transfusion reactions, red blood cell alloimmunization, and hemolytic transfusion reactions [59]. There has been a significantly decreased risk of infectious disease transmission due to donor testing and pathogen-reduction measures. Noninfectious complications have become more prominent. The FDA reports TRALI as the primary cause of transfusion-related fatalities, responsible for 34% (64/186) of deaths from 2012 to 2016, while TACO accounted for 30% (56/186) of such deaths in the same period [60]. It is therefore advisable to exercise caution and restrict the utilization of transfusions until further research can inform better mitigation strategies [59].

### 3.2. Cryoprecipitate 

Cryoprecipitate is obtained by thawing FFP (1–6° centigrade) and extracting the liquid portion of the plasma. Once the liquid component is removed, the remaining precipitate comprises factor XIII, Factor VIII:C, fibrinogen, von Willebrand factor (VWF), and fibronectin. It is subsequently frozen and stored at temperatures of −25 °C or lower for up to 36 months [61]. The remaining plasma, after removing the above factors, is called cryo supernatant or cryo-poor plasma. The only theoretical therapeutic application for cryo-poor plasma is in thrombotic thrombocytopenic purpura, which may require the transfusion of von Willebrand factor [62]. However, this has not been validated in clinical trials.

In the ICU, cryoprecipitate is recommended in situations of low or dysfunctional levels of fibrinogen with bleeding. Examples include postpartum hemorrhage (fibrinogen levels less than 200 mg/dL), liver disease (fibrinogen levels less than 150 mg/dL), and disseminated intravascular coagulopathy (fibrinogen levels less than 100 mg/dL) [63,64,65]. A multidisciplinary Guideline Development Group (GDG) recommends transfusing cryoprecipitate in cases of active bleeding and coexisting acquired fibrinogen deficiency [61]. Recommendations are made despite the absence of robust evidence as the benefits of reducing bleeding and preventing clinical deterioration offset the risks associated with transfusion. The risks of cryoprecipitate transfusions mirror those of FFP, and considering cryoprecipitate contains fibrinogen in a much smaller volume than FFP, it is advantageous in minimizing the risk of conditions like TACO in comparison [61]. A suitable dose for an adult is between 5 and 10 units or one to two 5-unit “pools” and is typically administered in a volume ranging between 50 and 200 mL. This dose contains about 2–2.5 g of fibrinogen and can raise the fibrinogen concentration to 70–100 mg/dL in an average individual [66]. 

## 4. Platelet Transfusion

Thrombocytopenia is defined as platelet levels less than 150 × 10^9^/L and less than 100 × 10^9^/L in the intensive care unit (ICU) setting [67,68]. The absolute platelet count by itself is, at times, insufficient. The rate of decline in platelet count can be significant. A drop in platelet count > 50% from baseline could be a normal finding after cardiac surgery; however, it is likely a reflection of an ongoing pathology in a critically ill patient [69]. Bedside platelet function studies are unavailable, and therefore, the platelet count becomes an important factor in the management of thrombocytopenia. In studies, the prevalence of thrombocytopenia in the ICU ranges from 13% to 60%. At the time of ICU admission, thrombocytopenia was noted in 8.3–67.6% of patients; 13.0–44.1% of patients developed thrombocytopenia throughout their stay in the ICU [70]. Several studies have reported that thrombocytopenia carries a poor prognosis in critically ill patients.

A study by Nijsten et al. shows that ICU survivors had a mean daily platelet count five times higher than non-survivors (30 ± 46 × 10^3^/mm^3^/day vs. 6 ± 28 × 10^3^/mm^3^/day (*p* < 0.001)) [71]. In a large study including forty ICUs, Akca et al. report that patients with persistent thrombocytopenia at two weeks of ICU stay had worse mortality than patients with improved counts (66% vs. 16%; *p* < 0.05) [72]. Many conditions in the ICU can predispose to low platelet counts, such as the use of circuits such as ECMO, invasive catheters, disseminated intravascular coagulation, multiple failures of organs, and post-cardiac resuscitation. A rapid decline in platelet counts should raise suspicion of drug-induced immune thrombocytopenias such as heparin-induced thrombocytopenia (HIT).

A unique clinical situation is where thrombocytopenia is associated with thrombosis. Many conditions such as DIC, heparin-induced thrombocytopenia (HIT), thrombotic thrombocytopenic purpura (TTP), and antiphospholipid syndrome (APS) feature such a presentation. In general, the transfusion of platelets is indicated only if there is severe bleeding, and specific treatment is encouraged. Expert consultation with a hematologist can be beneficial [70].

Indications for platelet transfusions in the ICU could be therapeutic or prophylactic. In a recent non-inferiority study published in the *New England Journal of Medicine*, patients with a platelet count of 10,000 to 50,000 per cubic millimeter undergoing central venous catheter placement without prophylactic platelet transfusion had more bleeding events [73]. According to the AABB (formerly known as the American Association of Blood Banks) and British Hematology Society (BSH), prophylactic transfusion is recommended at a platelet count of 10 × 10^9^/L or less in the setting of therapy-induced thrombocytopenia [74]. Table 4 lists indications for prophylactic platelet transfusion before procedures [74,75]. Mostly, these are expert opinions, with very few randomized studies supporting such recommendations.

## 5. Special Circumstances

### 5.1. Transfusion in Trauma Patients

Blood transfusion in trauma is a life-saving intervention that addresses severe hemorrhage, a leading cause of preventable death globally. It serves two crucial purposes: replenishing lost blood volume to maintain tissue oxygenation and restoring coagulation factors to prevent uncontrolled bleeding by promoting hemostasis. Traumatic hemorrhage leads to coagulopathy because of hemodilution, hypothermia, acidosis, consumption of clotting factors, and increased fibrinolysis. RBC, FFP, platelets, and cryoprecipitate are vital components in trauma resuscitation. RBCs restore oxygen-carrying capacity, while FFP, cryoprecipitate, and platelets replenish critical clotting factors [76].

Transfusion in trauma is based on balanced resuscitation strategies. Currently, the established transfusion regimen includes plasma:platelet:RBC ratios of 1:1:1 to 1:1:2 [77,78]. The landmark PROPPR trial, a multi-center, randomized trial, compared plasma:platelet:RBC ratios of 1:1:1 to 1:1:2, and there was no difference in overall mortality, but patients in the 1:1:1 group achieved hemostasis and had fewer death in first 24 h from exsanguination [79]. Another trial, “ROMMTT”, also evaluated the component ratios. They showed a 3–4 times higher mortality when ratios of plasma to RBCs and platelets to RBCs were below 1:2 compared to ratios above 1:1 [80].

Similarly, a retrospective review of over 9215 trauma patients receiving massive transfusion found that RBC: platelet transfusion ratios were unbalanced more frequently than RBC: plasma. Higher platelet: RBC transfusion ratios were associated with lower 24 h and 30-day mortality [81]. A disproportionately large volume of RBC without additional component replacement causes dilutional coagulopathy [82]. The American College of Surgeons also recommends a transfusion ratio of plasma and RBCs between 1:1 and 1:2 and transfusing one single donor apheresis or random donor platelet pool for each 6-unit RBC. In emergent trauma where crossmatched blood cannot be obtained within 15–20 min, patients can be transfused emergently with type-O blood. Type-specific blood can be safely administered even if the patient has not received uncross-matched red blood cells and is not associated with higher mortality [83]. The judicious use of diagnostic studies such as thromboelastography (TEG) or rotational thromboelastometry (ROTEM), along with therapeutic adjuncts, such as tranexamic acid (TXA), pro-thrombin complex concentrates, and activated factor VIIa, can help improve survival [82,84,85]. The CRASH 2 trial showed a decrease in mortality if TXA was given within three hours of trauma [86].

Permissive hypotension is a resuscitation strategy employed in hemodynamically unstable and actively bleeding trauma patients, involving lowering the mean arterial pressure (MAP) threshold below normal physiological levels with a goal of achieving source control of bleeding at the earliest moment. While it is considered beneficial for penetrating and blunt trauma, its efficacy in blunt trauma is uncertain, with reported poor outcomes and increased mortality rates due to tissue hypoperfusion [87]. However, it is contraindicated in traumatic brain injury (TBI) and traumatic spinal injury, where a systolic pressure above 80 mm Hg is recommended to maintain a cerebral perfusion pressure of at least 60 mm Hg [88]. The Brain Trauma Foundation advises maintaining systolic pressures above 90 mm Hg in TBI patients [89]. Ideal candidates are those without a TBI and a chronic history of hypertension. Various techniques exist for initial resuscitation during permissive hypotension, with studies favoring fluid resuscitation at a rate of 60 mL/kg/hr to maintain a MAP of 40–60 mm Hg [90]. The choice between crystalloid and colloid solutions is debated, with 0.9% normal saline showing some pro-coagulatory effects. Timing is crucial in trauma patient stabilization, with prehospital IV fluid administration improving survival, particularly in cases involving permissive hypotension [90]. Permissive hypotension prevents coagulation factor dilution, attenuates the vasodilatory effect, and prevents blood loss, reducing the need for other blood products and reducing health care expenditure [87,91].

In traumatic brain injury, the ideal transfusion strategy for anemia has been an unanswered question until recently. There is some consensus that hemoglobin less than 7 g/dL in patients who are critically ill necessitates a red blood cell transfusion; however, the exact threshold between 7 and 10 g/dL is still a very contentious issue [92]. Randomized control trial data demonstrate no difference in neurological or mortality outcomes between restrictive and liberalized arms but increased adverse effects, such as ARDS in the liberal arm [92,93]. Current clinical practice guidelines recommend a target Hb of 7–9 g/dL, and for TBI patients who show signs of cerebral ischemia, a target Hb level exceeding 9 g/dL is recommended [92,94].

### 5.2. Transfusion in Hematological Malignancy

Recent data have highlighted the significant burden of mortality and morbidity associated with ICU admissions in patients with hematological malignancy. Transfusion remains a cornerstone of supportive treatment to correct anemia, thrombocytopenia, and coagulopathies. Transfusion thresholds in this population often align with those established for general critical care patients. Short-term RBC transfusion is commonly used for transient anemia in hematological malignancies during induction chemotherapy or hematopoietic cell transplantation (HCT). A landmark randomized phase 3 trial had found that the restrictive threshold (transfuse if Hb < 7 g/dL) was non-inferior to the liberal threshold (transfuse if Hb < 9 g/dL) in patients with hematologic malignancy, requiring HCT, resulting in no significant differences in clinical outcomes [95].

Further, a randomized phase 3 trial found a single-unit RBC-transfusion strategy was found to be non-inferior to double units of RBC transfusion in terms of mortality and complications without increasing the total number of RBC transfusions required [96]. A conservative transfusion strategy is recommended. Similarly, a threshold of platelet < 10 × 10^9^/L was recommended for prophylactic platelet transfusion in hematologic malignancies undergoing treatment or HCT, per the 2018 American Society of Clinical Oncology guideline update [97].

Plasma and cryoprecipitate transfusion are indicated to correct coagulopathies in hematological malignancy conditions such as disseminated intravascular coagulation (DIC) mediated by acute promyelocytic leukemia (APL). Differentiation syndrome (DS) is associated with a higher risk of coagulopathy and hemorrhage, which can be seen in patients with APL undergoing induction therapy with all-trans retinoic acid (ATRA) and acute myeloid leukemia (AML) on IDH and FLT3 inhibitors. Patients with DS necessitate significantly higher use of plasma, platelet, and red blood cell transfusions during induction compared to those without DS [98].

To mitigate the risk of graft-versus-host disease (GVHD) due to transfusion in high-risk immunocompromised patients, including HCT recipients, individuals with Hodgkin’s lymphoma, and those undergoing intense immunosuppressive chemotherapy (fludarabine, antithymocyte globulin, and alemtuzumab), irradiation of the blood product is recommended [97].

### 5.3. Transfusion for Burns

Anemia is common in patients with burns exceeding 10% of the total body surface area (TBSA). The multifactorial pathogenesis of postburn anemia includes direct erythrocyte destruction, suppressed marrow response to erythropoietin, and frequent phlebotomy [99,100]. A retrospective study comparing the outcomes of liberal vs. restrictive blood transfusion in burn patients showed that patients in the restrictive group had better outcomes. On average, the hemoglobin triggering a transfusion was 7.1 g/dL in the restrictive group and 9.2 g/dL in the liberal group. Patients in the restrictive group had a lower mortality rate at 30 days (19% vs. 38%, *p* = 0.03) and overall in-hospital mortality (22% vs. 46%, *p* = 0.003) [101]. In a large multicenter retrospective study on the effect of blood transfusion on the outcome of major burn patients, non-survivors received more blood transfusions (14 units vs. 8.6 units, *p* < 0.05) than survivors [102]. The administration of erythropoietin has not been shown to decrease postburn anemia or the requirements for blood transfusion [103].

### 5.4. Transfusion in Cerebrovascular Disease

#### 5.4.1. Ischemic Stroke 

There has been some research into the relationship between hematocrit levels and stroke risk. Higher hematocrit levels (>50%) have been linked with larger infarctions and increased mortality. There exists an apparent “U-shaped” relationship suggesting that high and low hematocrit concentrations (<30%) may increase stroke risk according to some studies [104,105]. There does not seem to be sufficient evidence to specify a hemoglobin target or transfusion trigger; however, it is recommended to maintain an Hb level above 9 g/dL to ensure adequate oxygen delivery to the brain [94].

#### 5.4.2. Subarachnoid Hemorrhage 

Studies on patients with subarachnoid hemorrhage (SAH) have shown mixed outcomes, with some linking transfusion with poor outcomes [104]. Complications like cerebral vasospasm, infections, and thrombotic events have been reported, which could be related to alterations in blood rheology and storage-induced changes [104]. No definitive hemoglobin level has been determined, and red blood cell transfusions’ influence on SAH remains uncertain. However, it is recommended to target a Hb range of 8–10 g/dL in these patients [94].

## 6. Conclusions

In conclusion, administering blood transfusions in the ICU is crucial in managing critically ill patients. The ultimate decision to transfuse should always be based on the clinical context, carefully assessing the patient’s clinical condition, hemodynamic status, and laboratory parameters. While blood transfusions can be lifesaving in certain situations, it is equally important to balance the potential benefits with the risks associated with transfusion, including transfusion reactions and complications. Guideline-based treatment relating to transfusion should be adhered to when possible but with an understanding that evidence-based recommendations are limited, and clinical judgment should be applied carefully. Close monitoring of patients during and after transfusions is essential to identify adverse reactions and promptly ensure timely intervention. Additionally, efforts should be made to minimize the use of blood products through strategies such as restrictive transfusion thresholds and alternative therapies when appropriate, guided by sound evidence and clinical judgment. Small-volume phlebotomy tubes and closed-loop systems can be helpful in reducing the need for blood product transfusion.

## Figures and Tables

**Table 1:** Non-infectious adverse reactions based on time of occurrence.

Early Reactions (Occurs within 24 h)	Delayed Reactions (Occurs after 24 h)
Febrile non-hemolytic transfusion reactions (FNHTR)	Delayed hemolytic transfusion reaction (DHTR)
Acute allergic transfusion reactions including anaphylaxis	Transfusion-associated graft versus host disease (TA-GVHD)
Acute hemolytic transfusion reaction (AHTR)	Post-transfusion purpura (PTP)
Transfusion-associated acute lung injury (TRALI)	Hyper-hemolysis
Transfusion-associated circulatory overload (TACO)	

**Table 2:** Landmark trials in ICU patients for RBC-transfusion strategy.

Reference	Study Design	Patient Population	Restrictive Strategy	Liberal Strategy	Outcomes
Hebert et al. (TRICC trial) [8]	Multicenter RCT	ICU patients (N = 838)	7 g/dL	10 g/dL	No difference in overall 30-day mortality
Holst et al. (TRISS Trial) [7]	Multicenter RCT	Septic shock (N = 998)	7 g/dL	9 g/dL	No difference in 90-day mortality (43% vs. 45%, *p* = 0.44)
Walsh et al. (RELIEVE Trial) [9]	Multicenter Pilot RCT	Critically ill patients on mechanical ventilation (N = 100)	7 g/dL	9 g/dL	No significantly lower mortality with a restrictive strategy

**Table 3:** Societal guidelines for RBC transfusion in various clinical conditions.

Society Guidelines	Non-Bleeding Patients	Acute Bleeding Patients	Patients Admitted for Sepsis/Septic Shock	Trauma Patients	Patients with Underlying Heart Disease	Patients Undergoing Cardiac Surgery
AAST guidelines 2009 [21]	<7 g/dL	Restrictive	No cut-off mentioned	<7 g/dL	<8 g/dL	NR
ASH guidelines 2013 [22]	<7 g/dL	Restrictive	<10 g/dL in first 6 h of resuscitation	NR	<8 g/dL	NR
AABB guidelines 2023 [23]	≤7 g/dL	≤7 g/dL	NR	NR	<8 g/dL	≤8 g/dL
ESICM guidelines 2021 [24]	≤7 g/dL	Restrictive strategy	NR	NR	<8 g/dL	Restrictive strategy
Red Cross 2021 [25]	<7 g/dL	<7–8 g/dL	<7 g/dL	NR	<8 g/dL	<7.5 g/dL

AAST: American Association for the Surgery and Trauma; ASH: American Society of Hematology; AABB: Association for the Advancement of Blood & Biotherapies; ESICM: European Society of Intensive Care Medicine; NR: no recommendation.

**Table 4:** Prophylactic platelet transfusion threshold in microliters (µL) according to various guidelines prior to procedures.

	Society	American Association of Blood Banks (AABB)	British Society for Hematology (BSH)
Procedure			
Central venous lines		<20 × 10^9^/L	<20 × 10^9^/L
Lumbar puncture		<50 × 10^9^/L	<40 × 10^9^/L
Insertion/removal of epidural catheter		-	<80 × 10^9^/L
Percutaneous liver biopsy		-	<50 × 10^9^/L
Major non-elective surgery		<50 × 10^9^/L	<50 × 10^9^/L
CNS surgery/ophthalmic surgery		-	<100 × 10^9^/L

AABB: American Association of Blood Banks; ASCO: American Society of Clinical Oncology; BSH: British Society of Hematology; CNS: central nervous system; WR: weak recommendation; MR: moderate recommendation; SR: strong recommendation; NR: no recommendation.
